# Refractive and Aberration Outcomes after Customized Photorefractive Keratectomy in Comparison with Customized Femtosecond Laser

**Published:** 2015

**Authors:** Valleh Sajjadi, Mohammad Ghoreishi, Ebrahim Jafarzadehpour

**Affiliations:** 1 Department of Optometry, School of Rehabilitation Science, Iran University of Medical Sciences; 2 Department of Ophthalmology, School of Medicine, Esfahan University of Medical Sciences; 3 Department of Optometry, School of Rehabilitation Science, Iran University of Medical Sciences

**Keywords:** Customized Femtosecond Laser, Customized Photorefractive Keratectomy, Myopia, Spherical Aberration

## Abstract

To compare the refractive and visual outcomes and higher order aberrations in patients with low to moderate myopia who underwent customized photorefractive keratectomy (PRK) or femtosecond laser in situ keratomileusis (Femto-LASIK) this research performed. This study includes data of 120 consecutive eyes of 60 patients with myopia between -3.00 D and -7.00 D with or without astigmatism in two surgery groups: PRK and Femto-LASIK. Refractive, visual, and aberration outcomes of the two methods of surgery were compared after 6 months of follow-up. After six months of follow-up, sphere and cylinder were found significantly decreased and there was no statistically significant difference between the two groups. The mean of uncorrected distance visual acuity in LogMar format for the PRK and Femto-LASIK groups was -0.03±0.07 and -0.01±0.08, respectively, which was not significantly different between the two groups. Higher orders and spherical aberrations increased in both groups significantly, while total aberrations decreased in both groups. After surgery, no differences were observed between the two groups in the amount of aberrations. In conclusion, Both PRK and Femto-LASIK are effective and safe in correcting myopia. In this study PRK induced more spherical and higher order aberrations than Femto-LASIK.

## INTRODUCTION

Laser in situ keratomileusis (LASIK) is one of the most commonly used refractive surgery methods at present (1, 2). Myopia is the most prevalent refractive error in these surgeries (3). Flap creation methods have improved dramatically during recent years; from microkeratome instruments in past (2), to femtosecond laser technology in present (4, 5). In this new method, a flap is created by a solid-state focusable photodisruptive laser that generates femtosecond (10-15 seconds) pulses at a near-infrared (1053 nm) wavelength and delivers these to the stromal tissue (6-10). Femtosecond lasers create uniform thickness flaps from the center to the periphery as compared to previous methods (11, 12). Therefore, many investigators suggest that this method causes less damage to the corneal tissue (13), and the flaps it creates reduce higher-order aberrations effectively (12, 14, 15). A few studies showed no significant difference between Femto-LASIK and microkeratome in terms of safety or producing higher-order aberrations (5, 16). However, customized ablation methods improve post-operative contrast sensitivity in comparison with conventional methods (17, 18). They also induce fewer spherical aberrations, thereby decreasing halo or reducing difficulties in night driving for patients (18). The importance of correcting eye aberrations, especially spherical aberrations, is related to the retinal image quality because these aberrations reduce the quality of retinal images and subsequently reduce the quality of vision (19). Both customized LASIK and customized photorefractive keratectomy (PRK) can reduce aberrations and thus increase the patient’s satisfaction after surgery. While some surgeons still believe that surface ablation may induce better quality of vision and reduce higher-order aberrations in comparison to LASIK, (20, 21) and also reduce the probability of ectasia (22), many studies have revealed the importance and effectiveness of femtosecond techniques in producing the best flap and reducing higher-order aberrations and improving the outcomes of surgery (2, 23-25). However, the differences between femtosecond LASIK (Femto-LASIK) and PRK have not been adequately assessed. In this study, we compared the difference in higher order aberrations, visual outcomes, and refractive outcomes 6 months after refractive surgery in patients who underwent customized PRK and those who underwent customized Femto-LASIK.

## MATERIAL AND METHODS

This study comprised 120 consecutive eyes of 60 patients (40 males and 80 females) who underwent refractive surgery. All patients provided informed consent. Patients who were eligible for the study were aged between 18 and 35 years (mean, 26.53 ± 3.95 years), had myopia between -3.00 and -7.00 diopters (D), had astigmatism less than half of the myopia, had stable refraction for at least 6 months, did not wear soft contact lens for 1 week and hard contact lens for 3 weeks before the baseline examinations. Other inclusion criteria were as follows: corrected distance visual acuity (CDVA) 20/30 or better, healthy central and peripheral retina and normal intraocular pressure, and central corneal thickness of at least 500 microns. Patients with systemic or ophthalmic abnormalities other than refractive error were excluded. Patients were randomly divided into two groups: PRK and Femto-LASIK. We used random-block method based on a computer-generated program to classify patients according to age and sex. Selection of laser surgery method was done by the surgeon using a sealed envelope which was opened just before surgery. Preoperatively, uncorrected distance visual acuity (UDVA), CDVA, manifest refraction, slit-lamp examination, dilated fundoscopy, corneal topography, aberrometry (Orbscan IIz, Bausch & Lomb), applanation tonometry, and ultrasound pachymetry were performed in all patients. Higher-order, spherical, and total aberrations were measured at 5- and 6-mm pupil diameters for all patients. Both groups came for follow-up examinations on 1 day, 5 days, and 6 months after surgery. In the PRK group, bandage contact lenses were removed on the fifth day. After 6 months, the following examinations were performed on the patients of both groups for evaluation of higher-order, spherical, and total aberrations: CDVA and UDVA measurements, manifest refraction analysis, biomicroscopic slit-lamp examination, corneal topography, and aberrometry. Refractive surgeries were performed with Technolas 217z laser platform (Bausch & Lomb). One single surgeon (Mohammad Ghoreishi) performed all surgeries in the Parsian Eye clinic, Isfahan. All the patients received tetracaine 1.0% three times before surgery for topical anesthesia. In the Femto-LASIK group, wavefront-optimized LASIK was performed using Femto-Second LDV (Ziemmer FEMTO LDV, Femtosecond Surgical Laser) for flap creation (26, 27). The laser energy was on nanojoule and the frequency was higher than 1 MHz and the spot overlap was 0.7 mm. Laser parameters were put as bellow: side cut angle at 30-degree, hinge of 0.6 mm, flap depth of about 100 microns, and 9.5 mm flap diameter. The targeted postoperative refraction for all patients was emmetropia.

Analysis Method

Statistical analyses were performed using SPSS software (version 21, SPSS, Inc.). We used paired t test and independent t test to compare preoperative and postoperative data within and between groups. A P value less than 0.05 was considered statistically significant.

## RESULTS

The study enrolled 120 eyes of 60 myopic patients (40 men, 80 women), of which 30 patients underwent PRK and 30 patients Femto-LASIK. Table 1 shows patients’ baseline characteristics. No statistically significant differences were observed in baseline characteristics between the PRK group and the Femto-LASIK group. Table 2 shows all aberrations in millimeters before the operation, and the values do not show statistical differences between the two groups. After 6 months, 83.05% of patients in the PRK group and 78.68% of patients in the Femto-LASIK group gained 20/20 or better.

**Table 1:** Baseline Characteristics by Groups

**Parameter**	**Entire patients**	**PRK**	**Femto-LASIK**	**P value**
**Age: Mean ± SD**	26.53 ± 3.95	26.67 ± 4.46	26.39 ± 3.40	0.69
**Mean sphere (D) ± SD**	-4.77 ± 0.94	-4.62 ± 0.92	-4.90 ± 0.95	0.10
**Mean cylinder (D) ± SD**	-1.01 ± 0.75	-0.87 ± 0.63	-1.13 ± 0.84	0.57
**Mean CDVA ( LogMar) ± SD**	0.00 ± 0.02	0.00 ± 0.01	0.00 ± 0.02	0.45

**CDVA = Corrected distance visual acuity; UDVA = uncorrected distance visual acuity**

**Table 2:** Baseline Aberrations Before Surgery

**Parameter**	**Entire patients**		**PRK**	**Femto-LASIK**	**P value**
**Mean HOA 5 mm (µm) ± SD**		0.26 ± 0.11	0.25 ± 0.11	0.27 ± 0.11	0.25
**Mean HOA 6 mm (µm) ± SD**		0.42 ± 0.17	0.42 ± 0.18	0.42 ± 0.16	0.81
**Mean Sph. A 5 mm (µm) ± SD**		0.02 ± 0.03	0.02 ± 0.04	0.01 ± 0.01	0.07
**Mean Sph. A 6 mm (µm) ± SD**		0.04 ± 0.06	0.04 ± 0.06	0.03 ± 0.06	0.58
**Mean total A. 5 mm (µm) ± SD**		4.75 ± 0.96	4.57 ± 0.91	4.92 ± 0.99	0.04
**Mean total A. 6 mm (µm) ± SD**		6.88 ± 1.40	6.67 ± 1.35	7.07 ± 1.43	0.11

**HOA = Higher order aberration; Sph A.= spherical aberration; A= aberration**

**Table 3:** Refractive outcomes 6 months postoperatively

**Parameter**	**Entire patients**		**PRK**	**Femto LASIK**	**P Value**
**Mean sphere (D) ± SD**		0.05 ± 0.45	0.05 ± 0.37	0.04 ± 0.52	0.88
**Mean cylinder (D) ± SD**		-0.39 ± 0.30	-0.41 ± 0.35	-0.38 ± 0.25	0.54
**Mean UDVA (LogMar) ± SD**		-0.02 ± 0.07	-0.03 ± 0.07	-0.01 ± 0.08	0.18
**Mean CDVA (LogMar) ± SD**		-0.05 ± 0.06	-0.05 ± 0.06	-0.04 ± 0.05	0.27

**CDVA = corrected visual acuity; UDVA = uncorrected distance visual acuity**

Moreover, 42.37% and 29.5% of patients gained 20/16 or better in the PRK group and the Femto-LASIK group, respectively. Two eyes lost two lines (3.38%) in the PRK group, but there was no vision loss in the Femto-LASIK group. The mean postoperative BCVA in the PRK group was -0.05 and that in the Femto-LASIK group was -0.04 in logMAR values. Table 3 shows postoperative refractive results of the two groups after 6 months. A significant decrease in the magnitude of sphere and astigmatism occurred postoperatively toward the target refraction of emmetropia, but the difference was not statistically significant between the two groups. We compared aberration data before and after surgery in each group separately, and results are shown in Table 4. In the Femto-LASIK group, higher-order aberrations in the 5-mm pupil and spherical aberrations in the 5-mm pupil did not show any significant differences. Total aberrations in the 5-mm and 6-mm pupils decreased significantly. In addition, higher-order aberrations and spherical aberrations in the 6-mm pupil increased significantly.

**Table 4:** Aberration Outcomes Six Months Postoperatively

**Parameter**	**Femto-LASIK**			**PRK**		
	**Before**	**After**	**P value**	**Before**	**After**	**P value**
**Mean HOA 5 mm (µm) ± SD**	0.27±0.1	0.26±0.1	0.52	0.25±0.11	0.29±0.11	0.03
**Mean HOA 6 mm (µm) ± SD**	0.42±0.16	0.51±0.19	0.01	0.42±0.18	0.57±0.23	0.00
**Mean Sph A. 5 mm (µm) ± SD**	0.01±0.01	0.01±0.02	0.58	0.02±0.04	0.03±0.07	0.38
**Mean Sph A. 6 mm (µm) ± SD**	0.03±0.06	0.06±0.06	0.00	0.04±0.06	0.10±0.14	0.00
**Mean total A. 5 mm (µm) ± SD**	4.93±0.98	0.61±0.25	0.00	4.57±0.91	0.57±0.23	0.00
**Mean total A. 6mm (µm) ± SD**	7.08±1.42	1.02±0.36	0.00	6.67±1.35	1.04±0.45	0.00

**HOA = Higher order aberration; Sph A.= spherical aberration; A= aberration**

**Table 5:** Mean of Aberrations After Six Months

**Parameter**	**PRK**	**Femto-LASIK**	**P value**
**Mean difference HOA 5 mm (µm) ± SD**	0.04±0.14	-0.01±0.16	0.06
**Mean difference HOA 6 mm (µm) ± SD**	0.14±0.26	0.09±0.27	0.26
**Mean difference Sph A. 5 mm (µm) ± SD**	0.00±0.07	0.00±0.03	0.55
**Mean difference Sph A. 6 mm (µm) ± SD**	0.06±0.13	0.03±0.08	0.12

**HOA = Higher order aberration; Sph A.= spherical aberration; A= aberration**

**Table 6:** Postoperative Aberrations

**Parameters**	**PRK**	**Femto-LASIK**	**P value**
**Mean HOA 5 mm (µm) ± SD**	0.29±0.11	0.26±0.1	0.13
**Mean HOA 6 mm (µm) ± SD**	0.57±0.23	0.51±0.19	0.10
**Mean Sph A. 5 mm (µm) ± SD**	0.03±0.07	0.01±0.02	0.40
**Mean Sph A. 6 mm (µm) ± SD**	0.10±0.14	0.06±0.06	0.81
**Mean total A. 5 mm (µm) ± SD**	0.57±0.23	0.61±0.25	0.08
**Mean total A. 6 mm (µm) ± SD**	1.04±0.45	1.02±0.36	0.06

**HOA = Higher order aberration; Sph A.=spherical aberration; A= aberration**

The difference in the magnitude of the postoperative increase in higher-order and spherical aberrations did not reach statistical significance in either group (Table 5). After 6 months of follow-up examinations, we compared the aberration data of the PRK group with that of the Femto-LASIK group. There were no significant differences between the two groups (Table 6).

## Discussion

**Figure 1:**
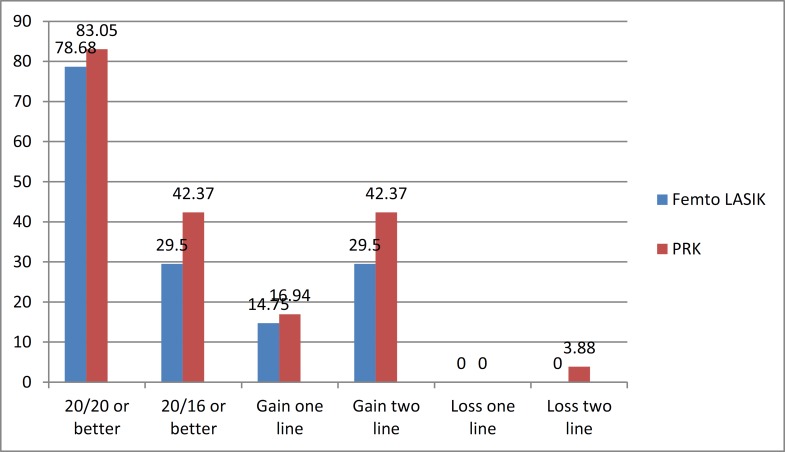
Visual Acuity Outcomes After Six Months

Our results confirmed the safety and effective power of both PRK and Femto-LASIK in correction of myopia. Many other studies reported the same result (8, 28-30). We followed up patients for 6 months because in the previous studies, refractive and visual results fluctuated during the first 3 months and led to different results. Therefore, we decided to follow up patients after 6 months to gain more stable results. In this study, more eyes achieved UDVA 20/20 or better after PRK than after Femto-LASIK (Figure 1).

Almahmoud et al. reported identical visual acuity of 20/20 or better in the PRK and Femto-LASIK groups after 3 months of follow-up (30). The percentage of patients with UDVA 20/20 or better in both groups was more in our study than in the study by Almahmoud et al. In the studies by Slade et al. and Durrie et al., the results were the same in both groups at 3 and 6 months’ follow-up (31, 32). Slade et al. in their next study reported that Femto-LASIK eyes demonstrated better results than those in PRK eyes in the first 3 months; however, at the 6-month follow-up, UCVA was similar in both groups (32). The difference was not statistically significant in our study, which was not in accordance with the findings in other investigations (32). It is clear that visual acuity after both methods of surgery led to the same results after 6 months according to the previous studies of Femto-LASIK in comparison to other methods of surface ablation (30-33). This is the main reason that investigators study contrast sensitivity and other subjective methods to discover more precise differences in the quality of vision instead of the quantity of vision. In our visual outcomes, the mean CDVA was better in the PRK group, but the mean CDVA in the previous studies was the same for the two groups after 3 months (30). In our study, 3.88% of the eyes in the PRK group lost two lines, while Femto-LASIK eyes did not show any visual loss. However, in the PRK group, 16.94% of the eyes gained one line and 42.37% gained two lines of visual acuity. In the Femto-LASIK group, 14.75% eyes gained one line and 29.5% gained two lines (Fig. 1). In the study by Almahmoud et al., patients in the PRK group had myopia between -1.00 and -8.00 D and those in the Femto-LASIK group had myopia between -1.00 and -9.50 D. The study also showed that no eyes lost any line in the PRK group, whereas 1.5% of the eyes lost one line in the Femto-LASIK group, with no one showing more than one line loss. In addition, 23% of PRK eyes and 16% of Femto-LASIK eyes gained one line (30). 

Thus, in the study by Almahmoud et al., similar to our study, visual outcomes were better in the PRK group. However, our patients gained more lines than their patients. Therefore, according to our study, improvement in visual acuity was better by both methods, which may be attributed to the different amounts of myopia in two studies. In the study by Debenito et al., the lost vision was the same in the LASEK and Femto-LASIK groups with equal risk of vision loss, and they proposed that both are safe methods (34).

In the study by Slade et al., Femto-LASIK eyes had better visual outcomes.(32) The reasons for visual loss in the PRK group can be (a) corneal haze, which was not seen in our patients or (b) increased higher-order aberrations, which were not significantly different between the two groups in our study. As shown in Table 3, visual and refractive outcomes in this study were the same in the two groups and did not show significant differences. Slade et al. and Durrie et al. demonstrated the same results (31, 32). The study by Debenito et al., which included high myopia, indicated that visual and refractive outcomes after 3 months were better in the Femto-LASIK group but the differences were not clinically important. In the next study by Debenito, the 6-month results showed more similarity between the groups (34). Similar results were achieved in the previous studies that evaluated low to moderate myopia and lower cases (31, 32). It should be mentioned that we used customized ablation in both methods of surgery, which shows an impressive decrease in aberration; however, Slade et al. used the conventional method.

**Figure 2:**
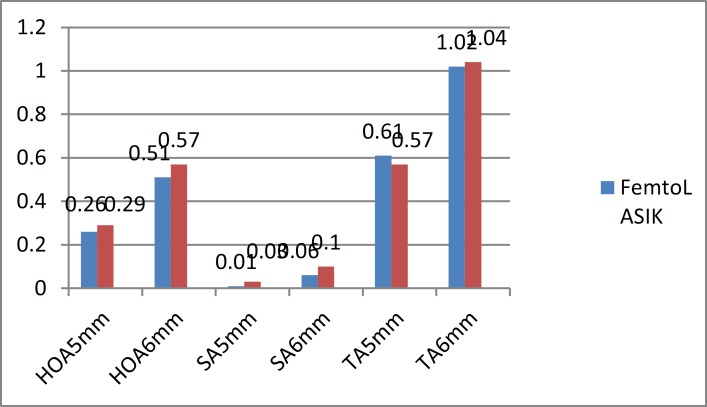
Aberration Outcomes After Six Months

Clinical experience and previously published research have indicated that non-optimal postoperative visual outcomes after refractive surgery are associated with an increase in ocular aberrations (35). To the best of our knowledge, none of the studies compared aberrations in different pupil sizes. However, some of them chose special sizes of pupils (30, 36). In our study, higher-order aberrations in the 5-mm pupil size did not show significant changes in the Femto-LASIK group, but the aberrations increased in the 6-mm pupil size in both groups and were higher in PRK patients. 

Thus, the increase in higher-order aberrations is lesser in the Femto-LASIK group than the PRK group. Almahmoud et al. showed the same results for the 5-mm pupil size (30). In a survey by Durrie et al., higher-order aberrations decreased in both groups after 6 months, but the reduction was greater in the PRK group; however, it was not significant (31). Durrie and associates chose one eye of a patient for PRK and the contralateral eye for LASIK, and this may explain the differences in two studies (31). Wallau et al. compared LASIK using microkeratome with PRK and after a 1-year follow-up, showed that higher-order aberrations are extremely high in the LASIK eyes (36). 

In our study, spherical aberrations in the 6-mm pupil size increased, which was more prominent in PRK patients. Thus, PRK increased spherical aberrations more than Femto-LASIK, which was the same as the result reported by Almahmoud (30). Slade et al. did not report any differences, whereas spherical aberrations showed better results in the PRK group in the study by Wallau et al., which was similar to our study (36).

Total aberrations decreased significantly in all patients, but the decrease was greater in Femto-LASIK eyes, thus confirming that Femto-LASIK was more effective in total aberration reduction. Slade et al. and Wallau et al. showed that total aberration reduction was more prominent in PRK eyes (36). In this regard, the Femto-LASIK flap may account for fewer induced aberrations. In particular, the corneal wound healing response after PRK has been found to be greater and longer than after LASIK (37). However, our study did not show any statistical significant differences between the two methods. Reduction in total aberrations may produce an effect on patient symptoms after surgery, which should be investigated in future studies. In our study, Femto-LASIK achieved better aberration outcomes than the PRK method; however, most of the studies showed better results for the Femto-LASIK group after 3 months, but after 6 months, the results became closer for both the groups (24). 

## CONCLUSION

The results of this study indicate that the visual outcomes were slightly better in the PRK group, but aberrations showed better results in the Femto-LASIK group.

## References

[1] Jabbur NS, Sakatani K, O'Brien TP (2004). Survey of complications and recommendations for management in dissatisfied patients seeking a consultation after refractive surgery. J Cataract Refrac Surg.

[2] Calvo Rn, McLaren JW, Hodge DO, Bourne WM, Patel SV (2009). Corneal Aberrations and Visual Acuity After Laser In Situ Keratomileusis: Femtosecond Laser Versus Mechanical Microkeratome. Am J Ophthalmol.

[3] Sugar A, Rapuano CJ, Culbertson WW, Huang D, Varley GA, Agapitos PJ (2002). Laser in situ keratomileusis for myopia and astigmatism: safety and efficacy: A report by the American Academy of Ophthalmology. Ophthalmology.

[4] Vengris M, Gabryte E, Aleknavicius A, Barkauskas M, Ruksenas O, Vaiceliunaite A (2010). Corneal shaping and ablation of transparent media by femtosecond pulses in deep ultraviolet range. J Cataract Refract Surg.

[5] Zhang Z-H, Jin H-Y, Suo Y, Patel SV, Montés-Micó R, Manche EE (2011). Femtosecond laser versus mechanical microkeratome laser in situ keratomileusis for myopia: Metaanalysis of randomized controlled trials. J Cataract Refract Surg.

[6] Kurtz RM, Horvath C, Liu HH, Krueger RR, Juhasz T (1998). Lamellar refractive surgery with scanned intrastromal picosecond and femtosecond laser pulses in animal eyes. J refract surg.

[7] Vaddavalli P, Yoo S (2011). Femtosecond laser in-situ keratomileusis flap configurations. Curr Opin Ophthalmol.

[8] Farjo AA, Sugar A, Schallhorn SC, Majmudar PA, Tanzer DJ, Trattler WB (2013). Femtosecond Lasers for LASIK Flap Creation: A Report by the American Academy of Ophthalmology. Ophthalmology.

[9] Santhiago MR, Wilson SE (2012). Cellular Effects After Laser In Situ Keratomileusis Flap Formation With Femtosecond Lasers: A Review. Cornea.

[10] Lubatschowski H (2008). Overview of commercially available femtosecond lasers in refractive surgery. J refract surg.

[11] Von Jagow B, Kohnen T (2009). Corneal architecture of femtosecond laser and microkeratome flaps imaged by anterior segment optical coherence tomography. J Cataract Refract Surg.

[12] Tran DB, Sarayba MA, Bor Z, Garufis C, Duh Y-J, Soltes CR (2005). Randomized prospective clinical study comparing induced aberrations with IntraLase and Hansatome flap creation in fellow eyes: Potential impact on wavefront-guided laser in situ keratomileusis. J Cataract Refract Surg.

[13] Kezirian GM, Stonecipher KG (2004). Comparison of the IntraLase femtosecond laser and mechanical keratomes for laser in situ keratomileusis. J Cataract Refract Surg.

[14] Perry S B (2004). Flap dimensions created with the IntraLase FS laser. J Cataract Refract Surg.

[15] Solomon KD, Donnenfeld E, Sandoval HP, Al Sarraf O, Kasper TJ, Holzer MP (2004). Flap thickness accuracy: Comparison of 6 microkeratome models. J Cataract Refrac Surg.

[16] Muñoz G, Albarrán-Diego Cs, Ferrer-Blasco T, García-Lázaro S, Cerviño-Expósito A (2010). Long-term comparison of corneal aberration changes after laser in situ keratomileusis: Mechanical microkeratome versus femtosecond laser flap creation. J Cataract Refract Surg.

[17] Kim T-i, Yang S-j, Tchah H (2004). Bilateral comparison of wavefront-guided versus conventional laser in situ keratomileusis with Bausch and Lomb Zyoptix. J Refrac Surg.

[18] Lee HK, Choe CM, Ma KT, Kim EK (2006). Measurement of contrast sensitivity and glare under mesopic and photopic conditions following wavefront-guided and conventional LASIK surgery. J Refract Surg.

[19] Applegate RA, Marsack JD, Ramos R, Sarver EJ (2003). Interaction between aberrations to improve or reduce visual performance. J Cataract Refract Surg.

[20] Chung S-H, Lee IS, Lee YG, Lee HK, Kim EK, Yoon G (2006). Comparison of higher-order aberrations after wavefront-guided laser in situ keratomileusis and laser-assisted subepithelial keratectomy. J Cataract Refrac Surg.

[21] Randleman JB, Loft ES, Banning CS, Lynn MJ, Stulting RD (2007). Outcomes of Wavefront-Optimized Surface Ablation. Ophthalmology.

[22] Perry S B (2007). Analysis of ectasia after laser in situ keratomileusis: Risk factors. J Cataract Refract Surg.

[23] Stonecipher K, Ignacio T, Stonecipher M (2006). Advances in refractive surgery: microkeratome and femtosecond laser flap creation in relation to safety, efficacy, predictability, and biomechanical stability. Curr Opin Ophthalmol.

[24] Ortiz D, Alió JL, Piñero D (2008). Measurement of corneal curvature change after mechanical laser in situ keratomileusis flap creation and femtosecond laser flap creation. J Cataract Refract Surg.

[25] Patel SV, Maguire LJ, McLaren JW, Hodge DO, Bourne WM (2007). Femtosecond Laser versus Mechanical Microkeratome for LASIK: A Randomized Controlled Study. Ophthalmology.

[26] Salomão MQ, Wilson SE (2010). Femtosecond laser in laser in situ keratomileusis. J Cataract Refract Surg.

[27] Soong HK, Malta JoB (2009). Femtosecond Lasers in Ophthalmology. Am J Ophthalmol.

[28] Cummings AB, Cummings BK, Kelly GE (2013). Predictability of corneal flap thickness in laser in situ keratomileusis using a 200 kHz femtosecond laser. J Cataract Refract Surg.

[29] Nassiri N, Safi S, Aghazade Amiri M, Sheibani K, Safi H, Panahi N (2011). Visual outcome and contrast sensitivity after photorefractive keratectomy in low to moderate myopia: Wavefront-optimized versus conventional methods. J Cataract Refract Surg.

[30] AlMahmoud T, Munger R, Jackson WB (2011). Effects of advanced surface ablations and intralase femtosecond LASIK on higher order aberrations and visual acuity outcome. Saudi J Ophthalmol.

[31] Durrie DS, Slade SG, Marshall J (2008). Wave front-guided excimer laser ablation using photorefractive keratectomy and sub-Bowman's keratomileusis: a contralateral eye study. J Refract Surg.

[32] Slade SG, Durrie DS, Binder PS (2009). A Prospective, Contralateral Eye Study Comparing Thin-Flap LASIK (Sub-Bowman Keratomileusis) with Photorefractive Keratectomy. Ophthalmology.

[33] Slade SG (2008). Thin-flap laser-assisted in situ keratomileusis. Curr Opin Ophthalmol.

[34] De Benito-Llopis L, Teus MA, Gil-Cazorla R, Drake P (2009). Comparison Between Femtosecond Laser-Assisted Sub-Bowman Keratomileusis vs Laser Subepithelial Keratectomy to Correct Myopia. Am J Ophthalmol.

[35] Moreno-Barriuso E, Lloves JM, Marcos S, Navarro R, Llorente L, Barbero S (2001). Ocular Aberrations before and after Myopic Corneal Refractive Surgery: LASIK-Induced Changes Measured with Laser Ray Tracing. Invest Ophthalmol Vis Sci.

[36] Wallau AD, Campos M (2009). One-year outcomes of a bilateral randomised prospective clinical trial comparing PRK with mitomycin C and LASIK. Br J Ophthalmol.

[37] Ma XH, Li JH, Bi HS, Zhou F, Li Y (2003). Comparison of corneal wound healing of photorefractive keratectomy and laser in situ keratomileusis in rabbits. Zhonghua Yan Ke Za Zhi.

